# WHEN NETWORKS FAIL: ISOLATION AND INDEPENDENCE AMONG OLDER ADULTS IN PUBLIC HOUSING

**DOI:** 10.1093/geroni/igz038.641

**Published:** 2019-11-08

**Authors:** Brooke Wagen, Whitney Williams, Jan Bennett, Elizabeth A Jacobs

**Affiliations:** 1 Dell Medical School at the University of Texas at Austin, Austin, Texas, United States; 2 none, New Mexico, United States; 3 The University of Texas at Austin Dell Medical School, Austin, Texas, United States

## Abstract

In the coming decades, the population of adults over 65 in the US will increase dramatically. Many older adults live at or below the poverty level, and the growing lack of affordable housing combined with fixed incomes promises to increase the number of older adults facing combined housing and health challenges. Despite their vulnerability, little is known about the lived experiences of older adults aging in place in public housing. We conducted semi-structured qualitative interviews with 27 older adults at two public housing sites in Austin, Texas to gain an understanding of their thoughts on health, aging, home, community, and problem solving. We conducted interviews in Spanish (n=10) and English (n=17) with 16 female and 11 male interviewees with a mean age of 71.7 years (range 65-85 years). We systematically coded transcribed interviews and used grounded theory to analyze the data. Participants described feeling isolated due to language barriers, cultural perceptions about neighbors, and previous problematic experiences with neighbors leading to intentional isolation for safety. Some, however, spoke of how they acted as community connectors or responded to connectors in the community in ways that reduced their isolation. Participants framed individual problem-solving and personal choices as central to health and wellness. Our findings suggest a way forward for housing authorities, communities, and health systems working together to provide services to these adults. Incorporating their points of view and even co-creating interventions to enhance their health and well-being will make these interventions more successful and welcome.

